# A Deep Learning Approach for the Assessment of Signal Quality of Non-Invasive Foetal Electrocardiography

**DOI:** 10.3390/s22093303

**Published:** 2022-04-26

**Authors:** Gert Mertes, Yuan Long, Zhangdaihong Liu, Yuhui Li, Yang Yang, David A. Clifton

**Affiliations:** 1 Department of Engineering Science, Institute of Biomedical Engineering, University of Oxford, Oxford OX1 2JD, UK; gert.mertes@ndph.ox.ac.uk (G.M.); jessie.liu@oxford-oscar.cn (Z.L.); david.clifton@eng.ox.ac.uk (D.A.C.); 2 Oxford Suzhou Centre for Advanced Research, Suzhou 215123, China; 3 Department of Cardiovascular Medicine, Wuhan Children’s Hospital (Wuhan Maternal and Child Healthcare Hospital), Huazhong University of Science and Technology, Wuhan 430015, China; doudou129129@126.com; 4 Department of Oncology, Central Hospital of Wuhan, Huazhong University of Science and Technology, Wuhan 430014, China; huihui090718@126.com

**Keywords:** foetal ECG, convolutional neural network, signal quality

## Abstract

Non-invasive foetal electrocardiography (NI-FECG) has become an important prenatal monitoring method in the hospital. However, due to its susceptibility to non-stationary noise sources and lack of robust extraction methods, the capture of high-quality NI-FECG remains a challenge. Recording waveforms of sufficient quality for clinical use typically requires human visual inspection of each recording. A Signal Quality Index (SQI) can help to automate this task but, contrary to adult ECG, work on SQIs for NI-FECG is sparse. In this paper, a multi-channel signal quality classifier for NI-FECG waveforms is presented. The model can be used during the capture of NI-FECG to assist technicians to record high-quality waveforms, which is currently a labour-intensive task. A Convolutional Neural Network (CNN) is trained to distinguish between NI-FECG segments of high and low quality. NI-FECG recordings with one maternal channel and three abdominal channels were collected from 100 subjects during a routine hospital screening (102.6 min of data). The model achieves an average 10-fold cross-validated AUC of 0.95 ± 0.02. The results show that the model can reliably assess the FECG signal quality on our dataset. The proposed model can improve the automated capture and analysis of NI-FECG as well as reduce technician labour time.

## 1. Introduction

Foetal cardiac monitoring is an important step in ensuring a positive outcome at birth and to identify factors that negatively affect the health status of the foetus. Early monitoring may prevent permanent damage to the foetus and intrauterine death [1,2,3,4,5]. The gold standard for foetal cardiac monitoring is invasive foetal ECG (I-FECG) using a trans-vaginal electrode placed on the scalp of the foetus [6,7,8]. While this provides a good-quality recording comparable with adult ECG due to the direct contact of the electrode with the foetus, it can only be used during labour and comes with additional disadvantages such as the risk of passing infection. Non-invasive methods are therefore preferable as they impose less of a burden on the mother and foetus and they can be used for long-term monitoring even outside of the hospital. Non-invasive monitoring methods used in clinical practice include non-invasive foetal electrocardiography (NI-FECG) [2,3,9,10,11,12,13,14], foetal echocardiography (FECHO) [15,16,17], foetal magnetocardiography (FMCG) [18,19,20] and cardiotocography (CTG) [21,22,23]. Of these methods, FECHO and FMCG can provide morphologically accurate FECG recordings with high SNR, but they require expensive hardware and skilled personnel, and are not suitable for long-term monitoring. CTG is a commonly used method in clinical practice that uses a single ultrasound transducer to measure the foetal heart rate. It is cheap and easy to use, but it only measures the heart rate time series. NI-FECG is a promising prenatal monitoring method that is cheap and easy to use and has the potential to make morphologically accurate recordings, but the signal needs to be processed in order to obtain the FECG. An additional downside of NI-FECG is that signal quality is dependent on the gestational period because the vernix caseosa electrically shields the foetus. As the vernix caseosa starts to dissolve, approximately from week 38, signal quality starts to improve. It therefore has a wider applicability towards the end of the third trimester [24].

**Figure 1:**
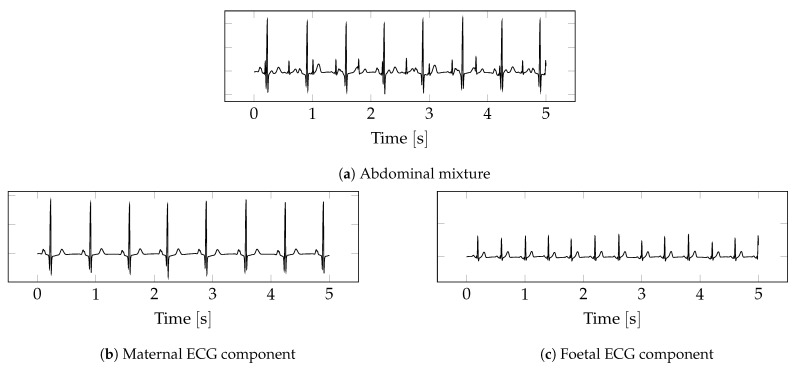
Example of NI-FECG. The abdominal mixture (**a**) contains both a maternal (**b**) and a foetal (**c**) component. The foetal component has a lower SNR than the maternal component. The figures depict an ideal case. In practice, the SNR is typically worse.

Non-invasive foetal electrocardiography (NI-FECG) is technologically similar to adult electrocardiography (ECG) [9]. Conventional ECG electrodes placed on the abdomen capture foetal cardiac activity. Figure 1a shows an example of a signal captured with NI-FECG. It is the sum of the maternal ECG (Figure 1b) and the foetal ECG (Figure 1c). It is impossible to measure either component individually using electrodes placed on the mother’s body, making it difficult to obtain a ground truth using non-invasive methods. CTG can be used as a reliable reference for the heart rate, but it does not provide the full signal. It is possible to capture the maternal ECG individually elsewhere on the body, but a simple subtraction of this signal from the abdominal mixture will not lead to the extraction of the FECG as the maternal component in the abdominal mixture is a non-linearly transformed version of the maternal ECG captured elsewhere on the body. The abdominal mixture is further corrupted by noise sources such as uterine and foetal movement. These noise sources are non-stationary, which often results in changing signal conditions throughout the recording. Signal quality is also dependent on the positioning of the foetus relative to the electrodes. Electrodes are typically placed in a way that covers a large area of the abdomen to increase the chances of capturing the foetal ECG in one of the leads. It is not uncommon for the foetus to move during a recording in such a way that causes the foetal component in the abdominal mixture to disappear entirely. These factors combined often result in a low and varying signal-to-noise ratio (SNR), making the extraction of FECG from the abdominal mixture a challenging task.

Because of the above-mentioned challenges, the majority of recent research efforts in this area are focused on the extraction of raw FECG by solving the signal-separation problem, or on the detection of foetal QRS (FQRS) complexes in the abdominal mixture [2,3,10,11,12,13,14]. The main limiting factor is that there is no general method that works for all NI-FECG signals, and models often need to be hand-crafted for each dataset [25]. Most works in this area also begin from a dataset in which it is known that the FECG is present with sufficient SNR. The question of how to deal with varying SNR remains largely unanswered by the current state of NI-FECG research.

Signal quality indices (SQI) have been shown to improve performance of signal-processing techniques for adult ECG and other physiological signals with a low or varying SNR. Li et al. [26] presented a set of SQIs derived from adult ECG and blood pressure (BP) measurements. The statistical characteristics of each waveform are used in combination with a Kalman filter to improve adult ECG heart rate (HR) estimation. The effect of including SQIs is an increased robustness of the HR estimation method in the presence of high levels of noise and during periods of extreme bradycardia and tachycardia. A F Pimentel et al. [27] presented a similar multimodal approach with SQIs derived from ECG and BP measurements to construct a HR estimation method using a hidden semi-Markov model. Johnson et al. [28] proposed an improved HR estimation method using an ECG SQI based on the agreement between two distinct R peak detectors. SQIs can also be used in a more straightforward way to determine the acceptability of collected ECG records in noisy environments, as shown by Clifford et al. [29]. Features were derived from the ECG to train a traditional supervised machine learning model to classify the signal quality. This method provides real-time feedback and prompts the user to make adjustments to the recording setup until the quality is sufficient so that an automated algorithm or medical expert can make a diagnosis. Similarly, Behar et al. [30] use SQIs and a traditional machine learning classifier to determine the signal quality during periods of arrhythmia, which leads to a reduction of false alarms.

Despite considerable advances in adult SQIs, work on a quality index for NI-FECG is sparse. The work by Andreotti et al. [31] was the first to propose an SQI specifically for NI-FECG. Using 45 features derived from 5 s abdominal mixture segments, a Naive Bayes (NB) classifier was trained to classify the foetal signal quality (FSQI) into five quality levels. The ground truth was annotated based on visibility of the FECG and perceived SNR. The model achieved a Cohen’s κ=0.44±0.03 (moderate) and Krippendorff’s coefficient α=0.65±0.04 (good). The posterior probability outputs of the NB model were then further used to improve a foetal heart rate (FHR) detection method based on Kalman filtering. This showed that—analogous to work on adult ECG—traditional machine learning methods can be used to estimate FSQI as well as improve performance of an upstream FECG application. To the best of our knowledge, it remains the only NI-FECG SQI in the literature to date. Deep learning approaches for foetal ECG processing are similarly sparse in the literature. Zhong et al. [32] presented a method to detect the foetal QRS in single-channel NI-FECG using a Convolutional Neural Network (CNN). Lee et al. [33] presented a similar approach on multi-channel NI-FECG. Zhong et al. [34] presented a method for the extraction of FECG using a residual convolution encoder–decoder network. Work on deep learning approaches for foetal SQIs is, to the best of our knowledge, absent in the literature.

The main contribution of this manuscript is to improve on the performance of the existing FSQI and eliminate the requirement of extensive feature engineering and hand-crafted methods. In [31], the majority of features are derived from the extracted FECG signal. Feature quality is therefore limited by the performance of the extraction algorithm, which can vary greatly depending on signal conditions [12,25]. We propose a method that estimates FSQI directly from the multichannel abdominal mixture by leveraging the automatic feature extraction properties of Convolutional Neural Networks (CNN). The remainder of this manuscript is structured as follows: first, the dataset and labelling procedure are discussed, followed by an overview of the proposed method, neural network architecture, and the evaluation methodology. The proposed deep learning model is benchmarked to the existing FSQI [31] with traditional machine learning models. After the reporting of the classification scores follows a discussion on the results and their clinical relevance. Finally, the conclusions are drawn.

## 2. Materials and Methods

### 2.1. Data and Labelling Procedure

**Figure 2:**
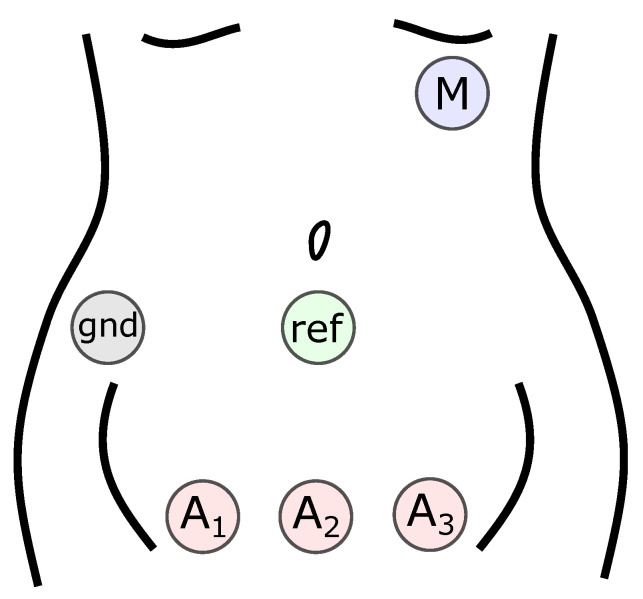
The NI-FECG electrode configuration used in this work.

The NI-FECG recordings used in this work were obtained from a private retrospective dataset collected at Wuhan Children’s Hospital, Wuhan, China from 2014–2019. Recordings were taken as part of a routine prenatal screening with pregnancies at various gestational ages. Each record consists of 1 maternal chest channel and 3 abdominal channels. Recordings are variable in length and typically in the range of 1–3 min. The sample rate is 1 kHz. Figure 2 shows the electrode configuration. The maternal chest lead (*M*) is placed over the 5th intercostal space on the left anterior axillary line (V5 position). The abdominal leads ($A_{1}$, $A_{2}$, $A_{3}$) are placed on the lower abdomen with the centre lead $A_{2}$ positioned over the pubic symphysis. The abdominal reference lead (ref) is placed over the fundus of the uterus. An additional ground lead (gnd) is placed level with the reference lead on the right mid-axillary line.

**Figure 3:**
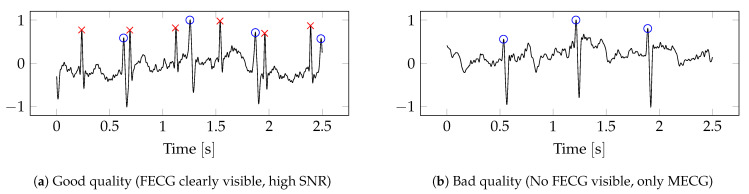
Examples of good- and bad-quality segments. Only the first abdominal channel is shown. Blue circle: maternal R peak, red cross: foetal R peak.

From the larger retrospective dataset, 100 recordings from unique singleton pregnancies were randomly selected for this work. Each recording was labelled as a whole and assigned a quality class label of good (1) or bad (0). Preference was given to recordings with a consistent perceived signal quality to ensure correct labelling over the whole recording. Recordings with inconsistent signal conditions were discarded. The label was assigned by the study’s data engineers based on the visibility of the FECG and perceived SNR in the abdominal channels (hospital clinicians were consulted on the decision process). In the hospital, the main criterium for signal quality is the ability to manually measure the foetal heart rate (FHR), and this approach was copied in the labelling process. Recordings where the FECG was sufficiently visible in at least 1 abdominal channel to manually determine the FHR were labelled as good quality. When the FECG was not visible or had low SNR such that the FHR was no longer reliably measurable, the recording was labelled as bad. While this is a coarse annotation, it reflects the procedures in the hospital where the same visual inspection is done after each recording. This yielded 56 recordings of good quality and 44 recordings of bad quality, for a total of 50.5 min of good data and 52.1 min of bad data. Figure 3 shows an example of a single abdominal channel segment of good and bad data. The maternal R peaks are labelled with a blue circle, and the foetal R peaks are labelled with a red cross. A larger version of this plot showing all 4 channels can be found in Appendix A.

### 2.2. Data Preprocessing

The data were first normalised to a minimum and maximum amplitude of −1 and +1, respectively. The normalised output ${\bar{x}}_{n}$ for input signal $x_{n}$ is given by:

(1) $${\bar{x}}_{n}=2\;\cdot \;\frac{x_{n}-x_{min}}{x_{max}-x_{min}}-1$$

**Figure 4:**
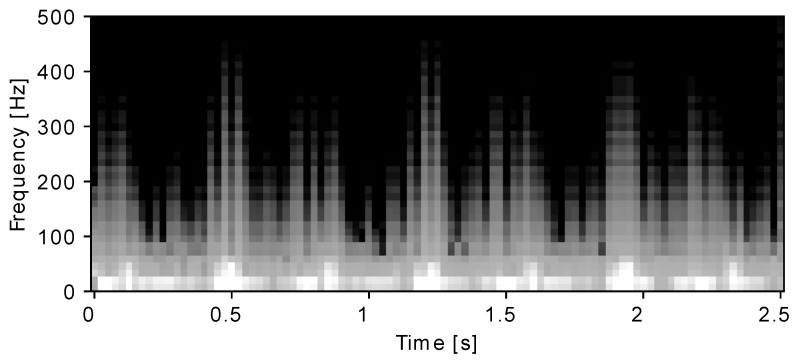
Time–frequency representation of an abdominal ECG segment (single-channel) with a good ground truth quality label.

Each normalised channel was filtered with a 3rd order Butterworth bandpass filter with a passband of 3–100Hz to suppress the baseline drift and unwanted high-frequency noise. Bandpass filtering is a commonly used preprocessing step for NI-FECG [2,3,10,11,12], and the passband of 3–100Hz is the same as what was used in [31]. Recordings were then segmented into 2.5 s windows with 0.1 s overlap. A window length of 2.5 s was used, which yields segments that typically contain 4–6 foetal R peaks and 3–5 maternal R peaks, which is sufficient to measure the heart rate. After segmentation, the dataset consisted of 1212 good segments and 1251 bad segments for a total of 2463 (a segment contains the maternal channel and 3 abdominal channels). Using the short-time Fourier transform (STFT), each channel was converted into a two-dimensional (2D) time-frequency representation. An 80-point Fourier transform with an equal length Hamming window and a time-step of 25 were used. The output image has a dimension of 40 × 126, and its values were scaled to a standard greyscale range of [0, 128]. Figure 4 shows an example of a single-channel segment of NI-FECG (with a good-quality ground truth label). The large maternal R peaks are visible with the smaller foetal R peaks in between.

### 2.3. Signal Quality Prediction

**Figure 5:**
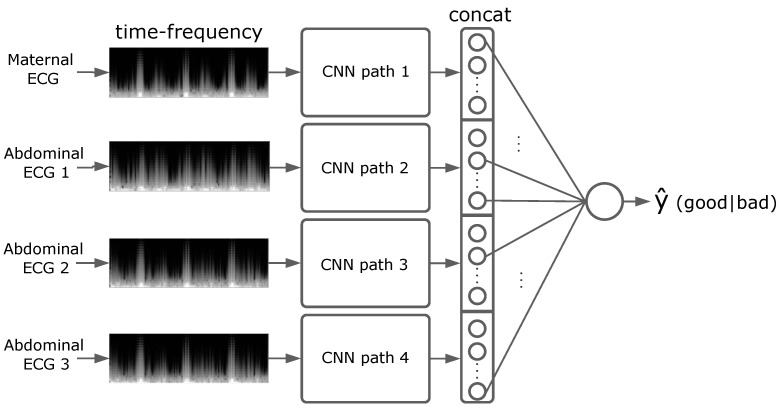
Neural network architecture for NI-FECG signal quality prediction.

Figure 5 shows the high-level neural network architecture. The network consists of 4 parallel convolutional paths, one for each ECG channel. The network is fed with the time-frequency image segments of each channel (2.5 s per segment). The CNN outputs its feature maps to a fully connected layer that is flattened and concatenated with the other CNN outputs. This is fed into a single output neuron with sigmoid activation to produce the predicted class probabilities $\hat{y}$ for the given input segment. The loss function for the network is binary cross-entropy, and the optimiser is Adam with lr=0.0005. The model was implemented using Keras v2.2.4 on TensorFlow v2.0.0.

**Figure 6:**
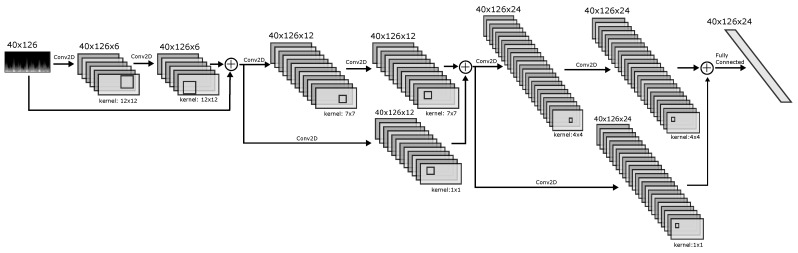
Detailed architecture of the CNN path for one channel. The network contains four of these in parallel.

Figure 6 shows the detailed architecture of the CNN path for a single channel. It consists of 3 equal pairs of Conv2D layers (filter and kernel sizes are shown in the figure). To minimise the loss of gradients during back-propagation, bypass connections are placed around each pair of Conv2D layers. The input of each bypass connection is summed with the output of every second Conv2D layer, and the sum is used as the input for the next Conv2D layer. To match the shape of the bypass connection to the output it is being summed with, a Conv2D layer with kernel of 1 × 1 and a matched filter size is placed in the bypass path. The last layer is a fully connected layer with 24 neurons. The output of this layer is flattened and concatenated with the other channels’ output, as shown in Figure 5. The stride for all Conv2D layers in the network is 1 × 1, and the activation is ReLu. Batch normalisation is performed after each Conv2D layer (not shown in the figure). A diagram showing the full network overview can be found in Appendix A.

### 2.4. Performance Evaluation

**Figure 7:**
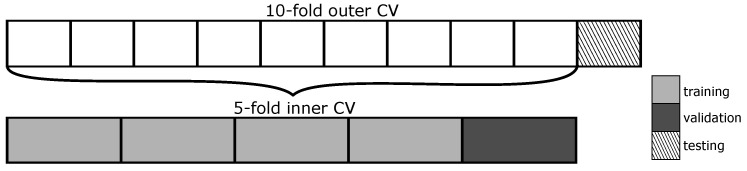
Illustration of the nested cross-validation procedure.

Nested 10-fold cross-validation (CV) was used for training and testing with a 5-fold inner CV for validation. Figure 7 illustrates the nested CV procedure. The entire dataset was first partitioned into 10 equally sized folds. Stratification was used to ensure an equal distribution of good and bad labelled data in each fold. The outer CV loop began by holding out one fold for testing while the remaining folds move on to the inner CV loop. The inner loop partitioned these data into 5 equally sized folds. One was held out for validation, and the remaining folds were used for training. The neural network was trained for 200 epochs with a batch size of 28 and evaluated on the validation fold at each epoch. The layer weights of the epoch with the highest area under the receiver operating curve (AUC) were stored. The inner loop repeated itself until each fold had been used for validation once, and the layer weights with the highest AUC out of the 5 training runs were selected and loaded into the model. The inner loop was exited and the testing fold held out by the outer CV loop is evaluated on the trained model. The performance on the test fold was stored and this concludes one iteration of the outer CV loop. The outer loop repeated itself 10 times, rotating the testing fold each time. To minimise possible bias introduced by the partitioning of the data, the outer CV was repeated 100 times with randomised folds. The final model performance score was obtained by averaging the test results over all iterations of the outer CV (1000 total).

The proposed neural network was benchmarked to the existing NI-FSQI based on feature engineering and traditional machine learning [31]. Features were calculated from the extracted FECG signal, including a set of commonly used signal metrics such as standard deviation, skewness, kurtosis, and newly proposed metrics based on the morphology of the extracted FECG signal and its detected QRS complexes. FECG extraction from the abdominal mixture was done with the $TS_{pca}$ method from [35], which was also used in [31]. Table 1 shows the complete list of benchmark foetal SQI features. For the implementation details, refer to the articles cited in the reference column. Because performance of different QRS detectors can vary depending on signal conditions, five different detectors were applied (maxsearch, jqrs, P&T, gqrs, wqrs) and feature calculation was duplicated on each detector’s output. This resulted in a total of 45 features. The code for these features was made available as part of the open source *FECGSYN* toolbox [36]. This code was used to extract features from the normalised NI-FECG segments—the same segments that were used for the proposed neural network prior to taking the STFT (as discussed in Section 2.1 and Section 2.2). The extracted features were then used to train the following machine learning models: Naive Bayes (NB), Support Vector Machine (SVM) with linear and Gaussian kernel and Random Forest (RF). Scikit-learn v0.23.1 was used. The same nested cross-validation procedure with 10-fold outer and 5-fold inner CV, as outlined above, was performed. In the inner CV loop, model hyper-parameters were tuned using a grid search. The complete list of grid parameters can be found in Appendix A. The model was trained with all combinations of hyper-parameters defined by the grid and evaluated on the validation fold, and the best performing combination of hyper-parameters was stored. Analogous to the neural network evaluation strategy, the best-performing hyper-parameters out of the 5 inner CV iterations were loaded into the model before evaluating the trained model with the outer CV test fold. The nested CV procedure was repeated 100 times, and the average performance was calculated.

## 3. Results

Table 2 shows the results of the nested CV for the proposed neural network (CNN-FSQI) and the benchmark machine learning models (NB, SVM, and RF). The CNN-FSQI outperformed the benchmark on all metrics. While the RF and SVM_rbf_ had similar average precision as the proposed CNN-FSQI approach, accuracy and recall were lower for the former. The proposed model has a significantly higher recall than all the benchmarks.

Table 3 shows the performance of the proposed CNN-FSQI model when including or excluding the maternal channel. The same 10-fold nested CV procedure was used, but only a single repetition was performed and the folds were fixed between experiments.

## 4. Discussion

The results in Table 2 show that the proposed neural network can predict the NI-FECG signal quality with good performance. The CNN-FSQI achieves an average accuracy of 0.94±0.02 and outperforms the NB model as proposed in [31]. The authors in [31] state that one of the improvements to the NB model would be a feature-selection step, which was omitted because the focus was on the development of an NI-FECG SQI, rather than fine-tuning of the machine-learning method. In order to allow a direct comparison with the aforementioned work, feature-selection was also omitted in the benchmark model. However, it is implicitly performed in the RF model during construction of the trees, where it selects the optimal features at the leaves. While the RF outperforms the NB, the proposed CNN-FSQI still outperforms the RF on all metrics.

Table 3 shows that inclusion of the maternal channel has an insignificant impact on model performance. The initial hypothesis for including the maternal channel was that it could contain information about the overall signal conditions such as the correct or incorrect positioning of the electrodes, a shared noise component, etc. However, this is evidently not the case for the dataset, or the model does not have the capacity to pick it up. In future work, the model could benefit from a mechanism to learn this information (e.g., attention). In a hospital environment where the maternal channel is available, it can be included for a slight boost in performance, but in situations where the maternal lead is not available (e.g., home monitoring devices) it can be omitted with a minimal impact on performance.

On average, the model has a false prediction rate of 0.71 False Positive (FP) and 0.71 False Negative (FN) per minute of NI-FECG data (1 FP and 1 FN every 84.55 s or 34 segments). The confusion matrix with the total number of predictions over all repetitions of the CV procedure can be found in Appendix A. In the hospital where the data were recorded, routine recordings were typically 1–3 min in length and never more than 5 due to time and manpower constraints. The current FP and FN rate is low enough that for a typical recording the false detections can be cancelled out by a simple majority voting algorithm when determining the signal quality for the whole recording.

**Figure 8:**
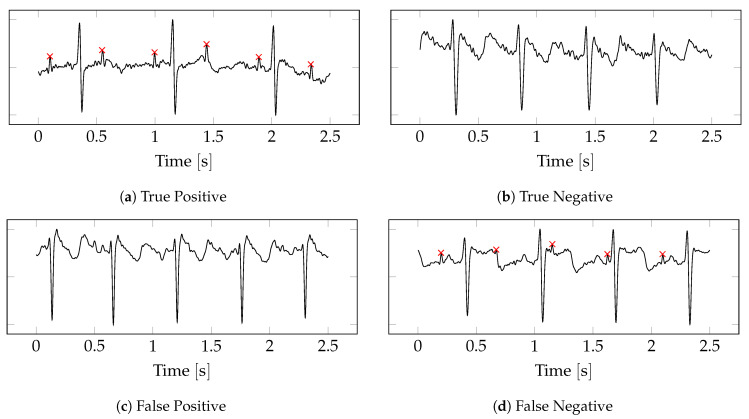
Examples of true and false predictions. Only the first abdominal channel is shown. The ground truth foetal R peaks are labelled with a red cross.

Figure 8 shows examples of true and false predictions (only the first abdominal channel is shown). Note that the good quality class is the positive label, and the bad quality is the negative label. For the True Positive (TP) case (Figure 8a), the foetal R peaks are easy distinguishable from the maternal signal. The True Negative (TN) case (Figure 8b) is similarly obvious. While the noisy component of the signal may contain the foetal ECG, it is indistinguishable by eye from the noise. The FP case (Figure 8c) appears to have been caused by the presence of several R peaks that appear to be of foetal origin. The position of the R peaks, however, cannot be clearly determined, and the segment was therefore given a ground truth label of bad. The FN is a borderline case (Figure 8d), and the model likely failed due to the low amplitude of the foetal R peaks. The segment has a ground truth label of good because the FHR can still be visually determined despite the low FECG amplitude.

The quality of the ground truth labels is an obvious limiting factor of the proposed method, since its performance can only be as good as its labels. Furthermore, the choice was made for a coarse labelling into two classes rather than a finer-grained labelling with multiple quality classes as performed in [31]. The reason for this was to simulate the same process that is currently followed in the hospital where the dataset was collected. During and after each recording, a technician performs a preliminary assessment of the signal in order to accept or reject it for diagnostic purposes. The main criterium is the ability to manually place markers on the foetal R peaks to measure the FHR. The same approach was taken during the ground truth annotation procedure in this work. Like in the hospital, this process can be subjective and there will inevitably be signals with a borderline quality where the ground truth is unclear. In future work, the method could benefit from the inclusion of a third unclear label for these borderline cases. This could also help to improve the interpretability of the results. The sigmoid activation of the model’s output layer produces a score between 0 and 1 that represents the model’s confidence that the input segment is of the good class. If the score is low, it is considered to be bad. Scores in the middle are those where the model cannot discriminate the class (e.g., the borderline cases). A threshold of 0.5 is used to assign the final binary class label, but the score can also be interpreted as a probability. With the current model and training data, however, the scores assigned to the test predictions are polarised; i.e., all scores are smaller than 0.01 or larger than 0.99, with no predictions in between. While this indicates that the model is good at discriminating the classes, it does not allow us to interpret the score as a probability, nor does it provide information about the borderline cases. In future work, interpretability could be improved if the model produced a score where values at the edges correspond to the bad and good classes, while unclear segments are located in the middle.

**Figure 9:**
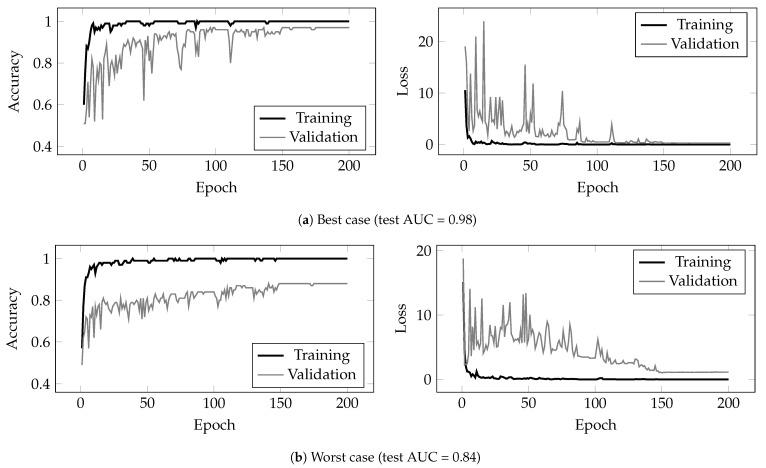
Training graphs for the best (**a**) and worst (**b**) repetitions of the CV procedure.

Another limitation of the work is the relatively small dataset. While the total number of 2.5 s segments was 2463, the number of subjects is only 100. Furthermore, the model’s capability to distinguish good from bad signals is limited by the available examples in the training set. For example, if a situation occurs that causes the signal quality to degrade that has not been seen during training, the model may not correctly identify it. In the current training dataset, most of the bad examples are signals where the foetal ECG has a low SNR, as this is the most common occurrence in the hospital. The number of signals that contain varying signal conditions throughout the recording such as uterine contractions or movements of the foetus is limited. In future work, these situations should be further examined. The cross-validation procedure does help to minimise bias introduced by partitioning of the data and illustrates the generalisation ability of the model. Figure 9 shows the accuracy and loss during training. Figure 9a shows the best repetition (based on test AUC) obtained over all rounds of the CV procedure (as outlined in Section 2.4). Figure 9b shows the worst repetition. Both the training and validation graphs converge and reach a plateau after 150 training epochs. The results indicate that the model generalises well on our dataset with minimal overfitting, but further clinical evaluation on more subjects is required.

In order to get an idea about the clinical applicability of the model, a clinician was asked to assign ground truth labels to a subset of the test segments. The clinician’s labels were then compared to the model’s predictions. This served two purposes: the first was to see whether the clinician’s ground truth labels are in agreement with those given by the study’s data engineer. The second was to see if the model’s assessment of the signal quality is in agreement with the clinician’s. The worst-performing fold of the outer 10-fold CV procedure based on AUC was chosen as the subset. This resulted in a subset where the model produced 41 TP, 49 TN, 14 FP and 4 FN. Of the TP and TN, 20 were randomly sampled from each without replacement. All FP and FN were included. The clinician labelled this subset as good, bad or unclear. The third unclear label was included here to get insight into the borderline cases. Table 4 shows the clinician’s ground truth labels against the True and False prediction cases based on the data engineer’s ground truth labels. For the TP and FN case, the clinician was in agreement with all cases. For the FP case, the clinician agreed nine times with the data engineer’s label that the prediction should have been bad. Only in one case did the clinician disagree. The remaining four cases were unclear. For the TN case, the clinician agreed seven times that the segment was bad and disagreed two times. The remaining 11 times were unclear. These results show that the clinician was in agreement with the model for the majority of predictions, which underlines the clinical applicability of the model. Furthermore, it shows that the model tends to classify the unclear cases as being of bad quality. When the goal is to reliably produce clinically useful recordings, it is preferable to reject the borderline case and redo the recording, rather than accept it only to find out later that the recording is useless.

The proposed method can be used to improve the quality of NI-FECG recordings in the hospital. Adult ECG recording sessions have a low turnaround time in the hospital in part due to the use of SQIs in the recording machine. The machine will alert the technician if something is wrong with the recording or if the quality is insufficient, and the technician only needs to perform a minimal visual inspection of the waveform to ensure its quality. For NI-FECG, on the other hand, the technician needs to perform a more time-consuming visual inspection and manually highlight the foetal R peaks to get the heart rate. Because the NI-FECG quality is dependent on foetal and electrode positioning, the technician should monitor the waveform at the start and during the recording to ensure that the foetal ECG is present. Due to manpower and high occupancy rates (in the case of the hospital where our data were recorded) this inspection is not always performed. This often results in waveforms where the foetal ECG is not visible. The proposed method can give an automated warning to the technician if the signal quality is insufficient, so that changes can be made to the electrode configuration or the recording can be stopped to not waste time and manpower. The method also has an application potential in NI-FECG home-monitoring devices, where the device has to work autonomously for longer periods of time. An SQI can tell the user if the device is worn correctly or if adjustments have to be made.

## 5. Conclusions

In this work, a deep learning approach for NI-FECG signal quality prediction was presented. The model was evaluated on a private dataset collected in the hospital and benchmarked to traditional machine learning methods. The proposed model outperformed the benchmark and results indicate that the model is in agreement with clinician-assigned ground truth labels. In future work, the model can be further improved by an improved labelling procedure with finer-grained labels and more clinician input, as well as expanding the dataset.

## Figures and Tables

**Table 1:** Benchmark foetal SQI features [31,37].

Cat.	SQI	Mult.	Description	Ref.
Time	stdSQI	no	standard deviation of signal: $std\left(x\left(t\right)\right)=\sqrt{E[{\left(x\left(t\right)-\bar{x}\left(t\right)\right)}^{2}]}$	[26,29]
	sSQI	no	third movement (skewness): $sSQI=\frac{E[{\left(x\left(t\right)\;-\;\bar{x}\left(t\right)\right)}^{3}]}{std{\left(x\left(t\right)\right)}^{3}}$	[26,29]
	kSQI	no	fourth moment (kurtosis): $kSQI=\frac{E[{\left(x\left(t\right)\;-\;\bar{x}\left(t\right)\right)}^{4}]}{std{\left(x\left(t\right)\right)}^{4}}$	[26,29]
Frequency	pSQI	no	relative power in the FQRS complex: $pSQI=1-\int_{5\mathrm{Hz}}^{15\mathrm{Hz}}{\left|X\left(f\right)\right|}^{2}df/\int_{5\mathrm{Hz}}^{45\mathrm{Hz}}{\left|X\left(f\right)\right|}^{2}df$, where X(f)=F(x(t)) is the Fourier transform of x(t).	[26,29]
	basSQI	no	relative power of baseline (bandwidth modified to [0,3] Hz to include most of the uterine contraction artefacts: $basSQI=\int_{0\mathrm{Hz}}^{3\mathrm{Hz}}{\left|X\left(f\right)\right|}^{2}df/\int_{0\mathrm{Hz}}^{100\mathrm{Hz}}{\left|X\left(f\right)\right|}^{2}df$)	[26,29]
Detection-based	bSQI	no	percentage of beats commonly detected by two different QRS detectors. The $F_{1}$ metric is used.	[11,26,29]
	iSQI	yes	percentage of beats detected on current lead that were detected on all other leads.	[26,29]
	rSQI	no	regularity of obtained FQRS intervals $rSQI=1-N_{out}/N_{d}$, where $N_{out}$ is the number of outliers (FHRV>30bpm) and $N_{d}$ the total number of detections in the segment.	[28,38]
	cSQI	no	morphology conformity measure for FQRS similarity. Negative correlations were set to zero.	[31,37]
	xSQI	no	extravagance of FQRS peaks compared to its surroundings	[31,37]
FECG-specific	mxSQI	yes	analogous to 1−xSQI considering the amplitude of MECG complexes residuals (100 ms window around MQRS reference annotations of ±50 ms) in comparison with surrounding extracted abdominal signals.	[31,37]
	mpSQI	yes	relative spectral power of the first five harmonics of the MHR ($mpSQI_{a}$) or all harmonics in the interval [0.5, 10] Hz ($mpSQI_{b}$)	[31,37]
	mcSQI	yes	spectral coherence calculated between available signals. Two variants are applied: $mcSQI_{a}$ uses MECG and FECG and $mcSQI_{b}$ abdFECG and FECG.	[31,37]
	miSQI	yes	similar to iSQI between current FQRS detection and MQRS reference: $miSQI=1-iSQI_{MQRS,FQRS}$, aims at exposing falsely detected MQRS residuals	[31,37]

Mult.: refers to the method requiring multiple channels or not (including MECG chest lead). Except for the time domain metrics, all outputs belong to R∈[0,1]. Cat.: category. Ref.: reference.

**Table 2:** Cross-validation results of the proposed model (CNN-FSQI) and the benchmark traditional machine learning models trained with the FSQI features from [31,37]. Bold shows the highest performance per column. [avg. ± std.].

	Accuracy	Precision	Recall	F_1_	AUC
CNN-FSQI	0.94 ± 0.02	0.94 ± 0.03	0.94 ± 0.03	0.94 ± 0.02	0.95 ± 0.02
NB	0.83 ± 0.01	0.88 ± 0.02	0.76 ± 0.02	0.82 ± 0.02	0.88 ± 0.01
SVM_linear_	0.81 ± 0.02	0.86 ± 0.03	0.73 ± 0.04	0.79 ± 0.03	0.88 ± 0.02
SVM_rbf_	0.87 ± 0.01	0.91 ± 0.03	0.81 ± 0.02	0.86 ± 0.01	0.92 ± 0.01
RF	0.86 ± 0.01	0.92 ± 0.03	0.77 ± 0.01	0.84 ± 0.01	0.92 ± 0.02

**Table 3:** Performance comparison of including or excluding the maternal channel in the proposed model (CNN-FSQI). Ten-fold cross-validation results from a single repetition are shown. [avg ± std].

Input	Accuracy	Precision	Recall	F_1_	AUC
*M* $A_{1}$ $A_{2}$ $A_{3}$	0.94 ± 0.02	0.94 ± 0.04	0.93 ± 0.06	0.93 ± 0.02	0.96 ± 0.02
$A_{1}$ $A_{2}$ $A_{3}$	0.92 ± 0.04	0.92 ± 0.05	0.93 ± 0.04	0.92 ± 0.04	0.93 ± 0.04

*M*: Maternal channel. $A_{i}$: Abdominal channel *i*.

**Table 4:** Number of ground truth labels given by the clinician compared to the evaluation based on the labels given by the study’s data engineer. Evaluation was done on the worst-performing fold of the nested 10-fold CV procedure.

	Good (Positive)	Bad (Negative)	Unclear
TP	20	0	0
FP	1	9	4
TN	2	7	11
FN	4	0	0

**Figure A1:**
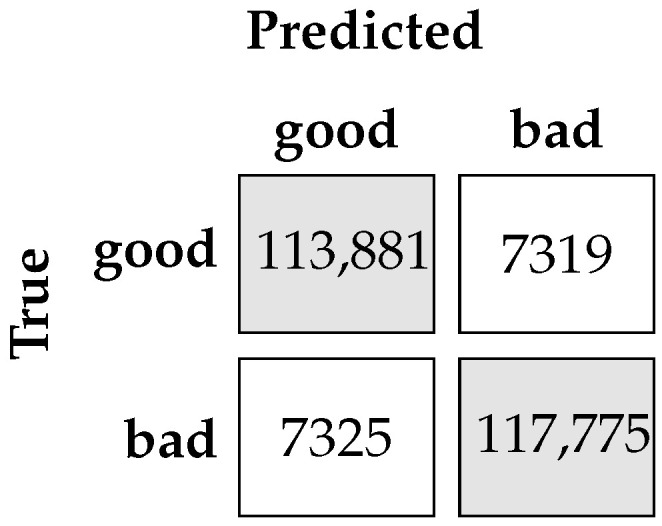
Confusion matrix of the CNN-FSQI cross-validation results. It shows the total number of predictions summed over all repetitions of the outer 10-fold CV.

**Figure A2:**
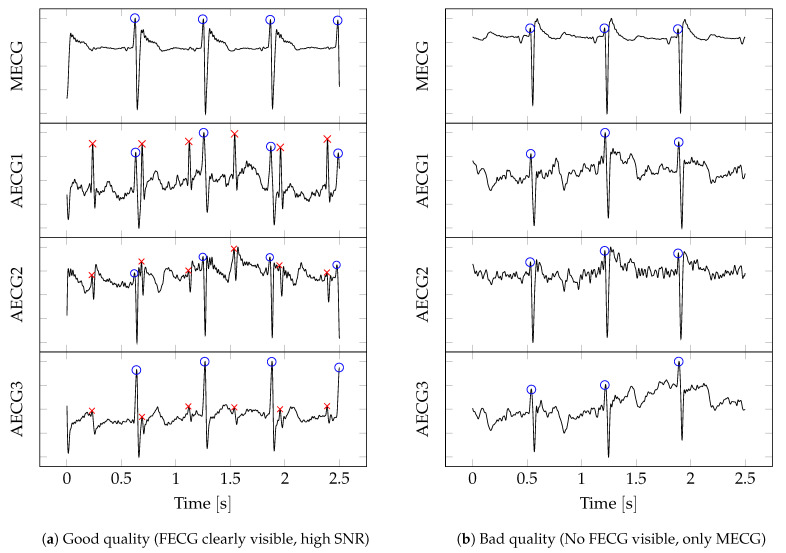
Examples of good- and bad-quality segments. Blue circle: maternal R peak, red cross: foetal R peak.

**Figure A3:**
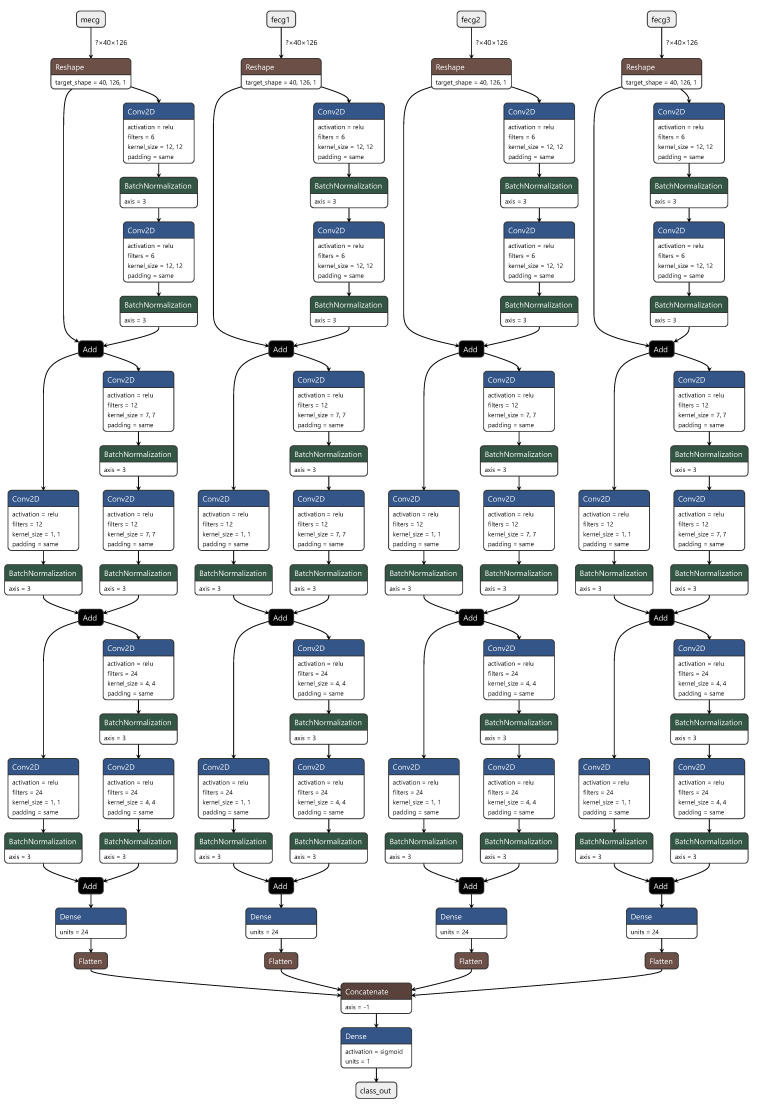
The full neural network architecture.

## Data Availability

The data used in this study are private.

## Appendix A

**Table A1:** Grid search parameters for the SVM with linear kernel.

Parameter					
C	0.1	1	10	100	1000

**Table A2:** Grid search parameters for the SVM with Gaussian (rbf) kernel.

Parameter							
C	0.1	1	10	100	1000		
γ	1	0.1	0.01	0.001	0.0001	*auto*	*scale*

**Table A3:** Grid search parameters for the RF.

Parameter												
*max_depth*	10	20	30	40	50	60	70	80	90	10	*None*	
*max_features*	*auto*	*sqrt*										
*min_samples_leaf*	1	2	4									
*min_samples_split*	2	5	10									
*n_estimators*	50	100	200	400	600	800	1000	1200	1400	1600	1800	2000
